# Dietary DHA/EPA ratio affected tissue fatty acid profiles, antioxidant capacity, hematological characteristics and expression of lipid-related genes but not growth in juvenile black seabream (*Acanthopagrus schlegelii*)

**DOI:** 10.1371/journal.pone.0176216

**Published:** 2017-04-21

**Authors:** Min Jin, Óscar Monroig, You Lu, Ye Yuan, Yi Li, Liyun Ding, Douglas R. Tocher, Qicun Zhou

**Affiliations:** 1 Laboratory of Fish Nutrition, School of Marine Sciences, Ningbo University, Ningbo, China; 2 Collaborative Innovation Center for Zhejiang Marine High-efficiency and Healthy Aquaculture, Ningbo University, Ningbo, China; 3 Institute of Aquaculture, School of Natural Sciences, University of Stirling, Stirling, Scotland, United Kingdom; Universitat Politècnica de València, SPAIN

## Abstract

An 8-week feeding trial was conducted to investigate the effects of dietary docosahexaenoic to eicosapentaenoic acid ratio (DHA/EPA) on growth performance, fatty acid profiles, antioxidant capacity, hematological characteristics and expression of some lipid metabolism related genes of juvenile black seabream (*Acanthopagrus schlegelii*) of initial weight 9.47 ± 0.03 g. Five isonitrogenous and isolipidic diets (45% crude protein and 14% crude lipid) were formulated to contain graded DHA/EPA ratios of 0.65, 1.16, 1.60, 2.03 and 2.67. There were no differences in growth performance and feed utilization among treatments. Fish fed higher DHA/EPA ratios had higher malondialdehyde (MDA) contents in serum than lower ratios. Serum triacylglycerol (TAG) content was significantly higher in fish fed the lowest DHA/EPA ratio. Tissue fatty acid profiles reflected the diets despite down-regulation of LC-PUFA biosynthesis genes, fatty acyl desaturase 2 (*fads2*) and elongase of very long-chain fatty acids 5 (*elovl5*), by high DHA/EPA ratios. Expression of acetyl-CoA carboxylase alpha (*accα*) and carnitine palmitoyl transferase 1A (*cpt1a*) were up-regulated by high DHA/EPA ratio, whereas sterol regulatory element-binding protein-1 (*srebp-1*) and hormone-sensitive lipase (*hsl*) were down-regulated. Fatty acid synthase (*fas*), 6-phosphogluconate dehydrogenase (*6pgd*) and peroxisome proliferator-activated receptor alpha (*pparα*) showed highest expression in fish fed intermediate (1.16) DHA/EPA ratio. Overall, this study indicated that dietary DHA/EPA ratio affected fatty acid profiles and significantly influenced lipid metabolism including LC-PUFA biosynthesis and other anabolic and catabolic pathways, and also had impacts on antioxidant capacity and hematological characteristics.

## Introduction

The C_18_ polyunsaturated fatty acids (PUFA) linoleic acid (LA, 18:2n-6) and α-linolenic acid (ALA, 18:3n-3) cannot be synthesized de novo in vertebrates and consequently they are regarded as dietary essential fatty acids (EFA) [1,2]. However, marine carnivorous fish have limited ability to convert LA and ALA into the physiologically important long-chain (C_20-24_) PUFA (LC-PUFA) such as eicosapentaenoic acid (EPA, 20:5n-3), arachidonic acid (ARA, 20:4n-6) and docosahexaenoic acid (DHA, 22:6n-3), and therefore these compounds must be supplied in their diet to ensure normal growth and development [3]. As marine ecosystems are naturally rich in LC-PUFA [4], adaptation to high dietary input of LC-PUFA in marine fish has been postulated as the evolutionary driver accounting for the loss of LC-PUFA biosynthetic capability in these species [3]. At a molecular level, specific deficiencies in one or more enzymes, namely fatty acyl desaturases (Fads) with and elongases of very long-chain fatty acids (Elovl), involved in LC-PUFA biosynthesis underpin the abovementioned limitation in the biosynthetic capability of marine fish [1]. Studies have shown that the black seabream *Acanthopagrus schlegelii*, a commercially important species for intensive culture in China, Japan, Korea and other countries in Southeast Asia [5–11], possesses a Fads2 with Δ6 desaturase activity [12], as well as an Elovl5 with high efficiency for elongation of C_18_ and C_20_ PUFA [13].

As DHA has important structural roles in biomembranes, especially in neural tissues such as brain and eye, where it is a major component of polar lipids [14,15], it is expected that DHA requirements are high in fast growing stages of development in order to satisfy the demands of rapidly forming tissues that accumulate DHA. While EPA has a major role as a precursor of highly bioactive compounds such as eicosanoids, it can also partly satisfy DHA requirements in species with adequate elongase and desaturase activities to convert EPA to DHA [1]. In addition, previous studies reported that the absolute requirement for n-3 LC-PUFA decreased with increased dietary DHA/EPA ratio [16,17]. Thus, in addition to the absolute dietary levels of DHA and EPA, their relative proportion is also an important aspect for consideration in feed formulations for fast-growing stages of fish [18]. The requirements of dietary DHA/EPA ratios for marine fish has been reported to range from 0.5 to 2.0 according to NRC [3].

Lipid metabolism involves anabolic (biosynthetic) and catabolic processes involving biochemical reactions catalyzed by key enzymes and regulated by, among others, transcriptional factors [19]. Acetyl-CoA carboxylase alpha (Accα) is a cytosolic enzyme that controls the production of malonyl-CoA and thus plays an important role in the biosynthesis of long-chain fatty acids [20–22]. Both 6-phosphogluconate dehydrogenase (6pgd) and glucose 6-phosphate dehydrogenase (G6pd) are key regulatory enzymes involved in NADPH production, essential for fatty acid biosynthesis [19,23]. Fatty acid synthase (Fas) catalyzes *de novo* fatty acid synthesis [24], whereas sterol regulatory element-binding protein-1 (Srebp-1) is a major regulator of fatty acid and lipid biosynthesis [25]. Among catabolic enzymes, lipoprotein lipase (Lpl) hydrolyzes triacylglycerols (TAG) in plasma lipoproteins and provides free fatty acids for either further storage in adipose tissue or oxidation in other tissues. Studies also confirm that Lpl plays a crucial role in regulating the content of body lipids [19,26]. Carnitine palmitoyltransferase (Cpt1) is regarded as the main regulatory enzyme in fatty acid oxidation catalyzing the conversion of cytosolic fatty acyl-CoA to fatty acyl-carnitine for entry into mitochondria [27,28]. Adipose triglyceride lipase (Atgl) and hormone-sensitive lipase (Hsl) are important enzymes involved in lipogenesis and lypolysis, respectively [29]. Peroxisome proliferator-activated receptor alpha (Pparα) can modulate expression of genes encoding several mitochondrial fatty acid-catabolizing enzymes in addition to mediating inducible mitochondrial and peroxisomal fatty acid β-oxidation [30].

Oxidative stress implies an increase in cellular production of free radicals, often resulting in cell and tissue damage [31,32]. There is concern that dietary PUFA, especially omega-3 LC-PUFA such as EPA and DHA, might increase oxidative stress [33,34] due to PUFA being susceptible to oxidation because oxygen easily attacks double bonds, producing lipoperoxides [35]. To protect cells and tissues from oxidative damage, animals including fish have endogenous antioxidant defense systems to help counteract the activity of free radicals. Protective enzymes include superoxide dismutase (SOD), which accelerates the dismutation rate of O_2_^-^ to H_2_O_2_ as a first line of enzymatic anti-oxidant defense, and glutathione peroxidase (GSH-PX) that reduces all organic lipid peroxides in a reaction that also requires glutathione (GSH) as a hydrogen donor [36–39]. Lipid oxidation products and their metabolites can be assayed in blood, urine and tissues as markers of endogenous lipid peroxidation/oxidative stress [40]. For instance, malondialdehyde (MDA), derived from the oxidation of fatty acids bearing more than two methylene interrupted double bonds [41], is an important metabolite derived from lipid peroxidation [42].

Overall, the dietary DHA/EPA ratio is of vital importance in fast growing stages of farmed fish and may affect key physiological and biochemical pathways that can ultimately compromise growth and normal development. Moreover, as far as we are concerned, no studies have measured lipid anabolism and catabolism genes to explore mechanisms related to the physiological effects of dietary DHA/EPA ratio in black seabream. Hence, the present study aimed to determine the effects of dietary DHA/EPA ratio (0.65–2.67) on growth performance, antioxidant capacity, fatty acid profiles and expression of some lipid-related genes of juvenile black seabream, *A*. *schlegelii*.

## Materials and methods

### Ethics statement

Animal experimentation in the present study was conducted in accordance with the Standard Operation Procedures (SOPs) of the Guide for Use of Experimental Animals of Ningbo University, and approved by the Institutional Animal Care and Use Committee of Ningbo University, China. Before handling and sacrificing, experimental fish were first anesthetized with tricaine methane sulfonate (MS-222).

### Diet preparation

Five isonitrogenous (~ 45% crude protein) and isolipidic (~ 14% crude lipid) diets were formulated to contain different ratios of DHA/EPA, with the diets named according to their respective DHA/EPA ratios, as “0.65”, “1.16”, “1.60”, “2.03” and “2.70” (Table 1). Casein, defatted fishmeal and soybean meal were used as protein sources, whereas palmitin (TAG containing palmitic acid as the sole fatty acid), soybean lecithin, and purified ARA, EPA and DHA were used as the main lipid sources. All ingredients except palmitin, ARA, EPA and DHA (details provided in Table 1) were purchased from Ningbo Tech-Bank Feed Co. Ltd., Ningbo, China. The fatty acid compositions of the diets are shown in Table 2. All dry ingredients were ground into fine powder with particle size < 177 μm, micro components such as minerals and vitamins premix were added followed by lipid and distilled water (35%, w/w). The ground ingredients were mixed in a Hobart type mixer and cold-extruded pellets produced (F-26, Machine factory of South China University of Technology) with pellet strands cut into uniform sizes (2 mm and 4 mm diameter pellets were prepared) (G-250, Machine factory of South China University of Technology). Pellets were steamed for 30 min at 90°C, and then air-dried to approximately 10% moisture, sealed in vacuum-packed bags and stored at −20°C until use in the feeding trial.

**Table 1:** Formulation and composition of the experimental diets (% dry matter).

Ingredient (%)	Dietary DHA/EPA ratio				
	0.65	1.16	1.60	2.03	2.67
Casein	16.23	16.23	16.23	16.23	16.23
Defatted Fishmeal^a^	18.00	18.00	18.00	18.00	18.00
Soybean meal	20.00	20.00	20.00	20.00	20.00
Wheat flour	30.00	30.00	30.00	30.00	30.00
Palmitin^b^	8.73	8.51	8.37	8.29	8.17
ARA-enriched oil^c^	1.00	1.00	1.00	1.00	1.00
DHA-enriched oil^d^	0.00	0.58	0.93	1.16	1.45
EPA-enriched oil^e^	1.34	0.98	0.77	0.62	0.45
Soybean lecithin	1.00	1.00	1.00	1.00	1.00
Vitamin premix^f^	0.50	0.50	0.50	0.50	0.50
Mineral premix^g^	1.50	1.50	1.50	1.50	1.50
Choline chloride	0.20	0.20	0.20	0.20	0.20
Ca (H_2_PO_4_)_2_	1.50	1.50	1.50	1.50	1.50
*Proximate composition (%)*					
Dry matter	92.20	92.60	92.40	92.50	91.00
Crude protein	45.46	45.10	45.50	45.67	45.09
Crude lipid	13.89	14.55	14.46	14.30	14.34
Ash	7.00	6.90	7.00	7.00	7.00

^a^ Defatted Fishmeal: 84% crude protein and 2.0% crude lipid

^b^ Palmitin: 97% of total fatty acids as palmitic acid methyl ester; Shanghai Yiji Chemical Co., Ltd., China.

^c^ ARA enriched oil: ARA content 40 mg g^−1^ oil, as triglycerides; Changsha Kenan Biotechnology Co., Ltd., China.

^d^ DHA enriched oil: DHA content, 437.7 mg g^−1^ oil; EPA content, 10.85 mg g^−1^ oil, as methyl esters; Jiangsu Tiankai Biotechnology Co., Ltd., China.

^e^ EPA enriched oil: EPA content, 501.3 mg g^−1^ oil; DHA content, 254.0 mg g^−1^ oil, as triglycerides; Hebei Kaiyuankangjian Biological Science and Technology Co., Ltd., China.

^f^ Vitamin premix based on Zhou et al. [9]

^g^ Mineral mixture (g kg^−1^ premix): FeC_6_H_5_O_7_, 11.43; ZnSO_4_·7H_2_O, 11.79; MnSO_4_·H2O (99%), 2.49; CuSO_4_·5H_2_O (99%), 1.06; MgSO_4_·7H_2_O (99%), 27.31; KH_2_PO_4_, 233.2; NaH_2_PO_4_, 228.39; C_6_H_10_CaO_6_·5H_2_O (98%), 34.09; CoC_l2_·6H_2_O (99%), 0.54. KIO_3_ (99%), 0.06; zeolite, 449.66.

**Table 2:** Fatty acid compositions (% total fatty acids) of the experimental diets with different dietary DHA/EPA ratios.

Name	Dietary DHA/EPA ratio				
	0.65	1.16	1.60	2.03	2.67
14:0	1.17	1.33	1.49	1.56	1.71
16:0	66.45	65.09	64.01	63.86	63.08
18:0	2.45	2.42	2.42	2.49	2.55
ΣSFA	70.07	68.84	67.92	67.91	67.34
16:1n-7	0.46	0.41	0.41	0.41	0.41
18:1n-9	5.83	5.56	5.45	5.31	5.26
20:1n-9	0.69	0.66	0.69	0.66	0.65
22:1n-9	1.37	1.43	1.49	1.49	1.48
24:1n-9	0.11	0.14	0.2	0.15	0.13
ΣMUFA	8.46	8.20	8.24	8.02	7.93
18:2n-6	7.85	8.01	8.08	8.06	8.14
20:4n-6	3.58	3.88	3.88	3.92	3.94
Σn-6PUFA	11.43	11.89	11.96	11.98	12.08
18:3n-3	0.73	0.76	0.78	0.78	0.84
20:5n-3 (EPA)	4.02	3.26	2.82	2.38	2.00
22:6n-3 (DHA)	2.62	3.77	4.51	4.82	5.33
Σn-3PUFA	7.37	7.79	8.11	7.98	8.17
n-3/n-6PUFA	0.64	0.66	0.68	0.67	0.68
n-3LC-PUFA	6.64	7.03	7.33	7.20	7.33
DHA/EPA	0.65	1.16	1.60	2.03	2.67

Some fatty acids, of which the contents are minor, trace amount or not detected, such as 12:0, 20:0, 20:2n−6, 20:3n−6, 22:5n−3, were not listed. DHA/EPA, 22:6n−3/20:5n−3; LC-PUFA, long-chain PUFA (C_20-24_); MUFA, monounsaturated fatty acids; PUFA, polyunsaturated fatty acids; SFA, saturated fatty acids.

### Feeding trial

Juvenile black seabream (initial weight 9.47 ± 0.03 g) were obtained from a local commercial hatchery at Xiangshan Bay, Ningbo, China. Prior to the experiment, the black seabream juveniles were acclimated for two weeks (26.0 ± 0.5°C) and fed on a commercial diet (45% dietary protein, 12% crude lipid, Ningbo Tech-Bank Corp.). A completely randomized trial design was implemented. A total of 600 black seabream juveniles were randomly allocated to 15 floating net cages (1.5 m × 1.5 m × 2.0 m) corresponding to triplicate cages of the five dietary treatments. Fish were hand-fed to apparent satiation twice daily at 5:00 am and 17:00 pm during eight weeks. During the experimental period, physico-chemical conditions including temperature 26.8–32.6°C, salinity (21–24 ‰), ammonia nitrogen (< 0.05 mg l^−1^) and dissolved oxygen (6.4–7.0 mg l^−1^) were monitored daily (YSI Proplus, YSI, Yellow Springs, Ohio, USA).

At the end of the feeding trial, fish were anesthetized with tricaine methane sulfonate (MS-222) at 100 mg l^−1^, the dose referred to Topic Popovic *et al*. [43]. Five fish from each cage (15 per treatment) were pooled (n = 3) and used for analyzing the proximal composition of whole body, where three fish (nine per treatment) were used to determine morphological parameters including condition factor (CF), viscerosomatic index (VSI), hepatosomatic index (HSI) and intraperitoneal fat (IPF) ratio. Muscle and liver samples were also collected and stored at −80°C until further analysis of fatty acid compositions (pools of 3 fish per cage, n = 3), antioxidant enzyme activity (pools of 3 fish per cage, n = 3) and liver gene expression (pools of 5 fish per cage, n = 3). Blood samples were taken from the caudal vasculature of 10 fish per cage and using 2 ml syringes. Among the 10 fish samples, eight were collected with non-heparinized syringes for essays on serum, whereas blood samples from 2 fish were taken using heparinized syringes for whole blood biochemical analyses.

### Proximate composition analysis

The crude protein, crude lipid, moisture and ash contents of diets and whole fish were determined according to the methods of the Association of Official Analytical Chemists (AOAC, 2006) [44]. Briefly, moisture content was determined by drying the samples to a constant weight at 105°C. Crude protein (N × 6.25) was determined via the Dumas combustion method with a protein analyzer (Leco FP528, St. Joseph, USA). Crude lipid was determined by the ether extraction method using the Soxhlet Method (Soxtec System HT6, Tecator, Sweden), and ash content was determined using a muffle furnace at 550°C for 8 h.

### Fatty acid composition

The fatty acid composition profiles of diets and fish tissues (liver and muscle) were determined as described by Zuo *et al*. [45] with minor modifications after tests to ensure that all fatty acids were esterified using the following procedures. Briefly, freeze-dried samples (liver samples ~80 mg and muscle samples ~120 mg) were added to a 12 ml volumetric glass tube with a screw top containing a teflon gasket. Three ml 1 M potassium hydroxide in methanol was added and the mixture incubated at 72°C for 20 min in a water bath. After cooling, 3 ml 2 M HCL in methanol was added and the mixture incubated at 72°C for a further 20 min. Finally, 1 ml hexane was added to the mixture, shaken vigorously for 1 min, and then allowed to separate into two layers. Fatty acid methyl esters were separated and measured by GC-MS (Agilent Technologies GC-MS 7890B-5977A, USA) with results presented as percentages of total fatty acids.

### Oxidation and antioxidant parameter assays

The blood was assayed within 24 h from collection after storage at 4°C, with serum collected by centrifugation at 956 g for 10 min at 4°C (Eppendorf, Centrifugal 5810 R, Hamburg, Germany). All serum samples were used undiluted, with the exception of samples for GSH-PX analysis, which were subject to two-fold dilutions prior use. Liver samples were homogenized in nine volumes (w/v) of ice-cold physiological saline 0.89% (w/v), and then centrifuged as above. The contents of MDA, GSH and protein, as well as enzymatic activities of GSH-PX and T-SOD, were determined in serum and liver homogenates using assay kits (Nanjing Jiancheng Bioengineering Institute, China). The liver homogenate concentrations in each index assay were as following: MDA (10%), GSH (10%), protein (1%, was diluted 10-folds), GSH-PX (10%) and T-SOD (0.125%, was diluted 80-folds). Prior calculations, the content of protein was adjusted according to different concentration of liver homogenate. All the content of MDA, GSH and protein, as well as enzymatic activities of GSH-PX and T-SOD, were calculated according to the manufacturer’s instructions.

### Haematological determinations

Total protein (TP), glucose (GLU), triacylglycerol (TAG) and cholesterol (CHOL) contents were measured in serum samples. Moreover, whole blood samples were used for red blood cell (RBC) and white blood cell (WBC) counts, hemoglobin (HGB) and hematocrit (HCT), with analyses carried out with an automatic blood analyzer (Hitachi 7600–110 Ltd, Japan) at Ningbo University Hospital.

### RNA extraction and reverse-transcriptase quantitative PCR

Gene expression was determined by reverse-transcriptase quantitative PCR (qPCR) as follows. Total RNA was extracted from the liver of juvenile black seabream using TRIzol reagent (Takara, Japan) according to the manufacturer’s instructions. Quantity and quality of isolated RNA were determined spectrophotometrically (Nanodrop 2000, Thermo Fisher Scientific, USA) and on a 1.2% denaturing agarose gel, respectively. The cDNA was prepared from 1000 ng of DNAase-treated RNA and synthesized using PrimeScript^™^ RT Reagent Kit with gDNA Eraser (Perfect Real Time) (Takara). The stability of potential references genes including *β-actin*, *gapd* and 1*8S rRNA* was tested using Bestkeeper [46]. The results confirmed that *β-actin* was very stable (stability value was 0.255) and was subsequently use as reference gene to normalize the expression levels of the candidate genes. Specific primers for the candidate genes including *accα*, *6pgd*, *g6pd*, *fas*, *srebp-1*, *lpl*, *cpt1a*, *atgl*, *hsl*, *pparα*, *elovl5 and fads2* used for qPCR were designed using Primer Premier 5.0 (Table 3). The primer specificity assay of the candidate genes was performed as described by Bustin *et al*. [47]. The primer specificity was checked by systematically running melting curve assays after the qPCR program, running the qPCR products on a 1% (w/v) agarose gel, and DNA sequencing technology (BGI, China). Amplification was performed using a quantitative thermal cycler (Lightcycler 96, Roche, Switzerland). The qPCR assays were performed in a total volume of 20 μL, containing 1.0 μL of each primer, 10 μL of 2× conc SYBR Green I Master (Roche), 2 μL of 1/5 diluted cDNA and 6 μL DEPC-water. The thermal-cycling conditions used for qPCR were as follows: 95°C for 2 min, followed by 45 cycles of 95°C for 10 s, 58°C for 10 s and 72°C for 20 s. Standard curves were generated using six different dilutions (in triplicate) of the cDNA samples, and the amplification efficiency was analyzed using the equation E = 10^(–1/Slope)^-1 [48]. The amplification efficiencies of all genes were approximately equal and ranged from 93 to 102%. In the present study, all the gene expression data were presented as relative gene expression with regards to the expression of the 0.65 DHA/EPA group (reference group). The relative expression levels were calculated using the 2^-ΔΔCt^ method as described by Livak and Schmittgen [49].

**Table 3:** Primers for real-time quantitative PCR for lipid related genes and *β-actin* of black seabream (*Acanthopagrus schlegelii*).

Gene	Nucleotide sequence (5’ –3’)	Size (bp)	GenBank reference or Publication
*accα* ^1^	F: AGTAGCCTGATTCGTTGGT	154	KX066238
	R: GATTGAGGAGTCTGTTCGC		
*6pgd* ^2^	F: GAAGGGCTTGCTGTTTGTT	101	KX066237
	R: GTGGCCAGGCCTCTATATG		
*g6pd* ^3^	F: TCTCGCCAAGAAGAAAATC	297	KX078573
	R: GCCAGGTAGAACAAACGGT		
*fas* ^4^	F: AAGAGCAGGGAGTGTTCGC	213	KX066240
	R: TGACGTGGTATTCAGCCGA		
*srebp*-*1*^5^	F: TGGGGGTAGGAGTGAGTAG	247	KX066235
	R: GTGAAGGGTCAGTGTTGGA		
*lpl*^6^	F: CTGCTACTCCTCTGCCCA	204	KX078571
	R: ACATCCCTGTTACCGTCC		
*cpala* ^7^	F: TGCTCCTACACACTATTCCCA	203	KX078572
	R: CATCTGCTGCTCTATCTCCCG		
*atgl*^8^	F: GCATCCAGTTCACCCTCAC	241	KX078570
	R: TTTGCCTCATCTTCATCGC		
*hsl*^9^	F: AGCAACTAAGCCCTCCCCATC	179	KX066236
	R: TCTTCACCCAGTCCGACACAC		
*pparα* ^10^	F: ACGACGCTTTCCTCTTCCC	183	KX066234
	R: GCCTCCCCCTGGTTTATTC		
*elovl5* ^11^	F: TCGTACTTCGGTGCCTCCCT	176	KU372149
	R: GGCCATATGACTGCAAATATTGTC		
*fads2* ^12^	F: AGCCAGGACCGAAATAAAA	113	KX058437
	R: AGGTGGAGGCAGAAGAACA		
*β-actin*	F: ACCCAGATCATGTTCGAGACC	212	Jiao *et al*. [50]
	R: ATGAGGTAGTCTGTGAGGTCG		AY491380

^1^ *accα*, acetyl-CoA carboxylase alpha;

^2^ *6pgd*, 6-phosphogluconate dehydrogenase;

^3^ *g6pd*, glucose 6-phosphate dehydrogenase;

^4^ *fas*, fatty acid synthase;

^5^ *srebp-1*, sterol regulator element-binding protein-1;

^6^ *lpl*, lipoprotein lipase;

^7^ *cpt1a*, carnitine palmitoyltransferase 1A;

^8^ *atgl*, adipose triglyceride lipase;

^9^ *hsl*, hormone-sensitive lipase;

^10^ *ppar*α, peroxisome proliferators-activated receptor alpha;

^11^ *elovl*5, elongase of very long-chain fatty acids 5;

^12^ *fads2*, fatty acyl desaturase 2.

### Calculations

The parameters were calculated as follows:

$$\text{Specific growth ratio}\left(\text{SGR},\;\%{\text{ day}}^{-1}\right)=100\times \left(\left(\text{Ln final body weight }\left(\text{g}\right)-\text{Ln initial body weight}\right)\;\left(\text{g}\right)\right)/\text{days}).$$

Feed efficiency(FE)=weight gain (g, wet weight)/feed consumed (g, dry weight).

Survival (%)=100×(final fish number/initial fish number).

$$\text{Condition factor }\left(\text{CF},\text{ g}/{\text{cm}}^{\text{3}}\right)=\text{Body weight }\left(\text{g}\right)\times 100/\text{body length }{\left(\text{cm}\right)}^{3}.$$

Viscerosomatic index (VSI, %)=100×(visceral weight/wet body weight).

Hepatosomatic index(HSI, %)=100×(liver weight/wet body weight).

Intraperitoneal fat ratio (IPF, %)=100×(intraperitoneal fat weight/wet body weight).

### Statistical analysis

Results are presented as means and SEM (number of replicates as indicated). The relative gene expression results (qPCR analyses) were expressed as mean normalized ratios (± SEM) corresponding to the ratio between the copy numbers of the target genes and the copy numbers of the reference gene, *β-actin*. The homogenity of variances (Levene’s test) were checked prior ANOVA tests. Effects of dietary DHA/EPA ratios were analyzed by one-way analysis of variance (ANOVA) followed by Tukey's HSD test at a significance level of *P* ≤ 0.05 (IBM SPSS Statistics 20).

## Results

### Growth performance and feed utilization

The impacts of dietary DHA/EPA ratio on growth performance and feed utilization are presented in Table 4. Specific growth rate (SGR), feed efficiency (FE) and survival did not show any statistical differences among dietary treatments (*P* > 0.05). Nevertheless, the highest value of SGR was found in fish fed a diet with an intermediate DHA/EPA ratio (1.60), whereas the diet with a DHA/EPA ratio of 2.03 showed the highest FE and survival (Table 4).

**Table 4:** Growth response, feed utilization and biometric indices of juvenile black seabream (*Acanthopagrus schlegelii*) fed diets containing different DHA/EPA ratios.

Parameter	Dietary DHA/EPA ratio											
	0.65		1.16		1.60		2.03		2.67		ANOVA *P*	F-value
	Mean	SEM	Mean	SEM	Mean	SEM	Mean	SEM	Mean	SEM		
IBW (g)	9.45	0.00	9.47	0.00	9.49	0.04	9.46	0.00	9.46	0.01	0.415	2.57
FBW (g)	36.87	0.26	36.78	0.01	37.36	0.16	36.77	0.24	36.90	0.11	0.194	1.86
SGR (%/d)	2.43	0.01	2.42	0.00	2.45	0.01	2.43	0.01	2.43	0.01	0.168	2.01
FE	0.72	0.01	0.72	0.01	0.71	0.01	0.73	0.01	0.70	0.02	0.294	1.43
Survival (%)	95.83	2.20	95.83	3.00	96.67	0.83	99.17	0.83	94.17	1.67	0.294	1.43
CF (g/cm^3^)	2.90	0.03	2.91	0.08	2.93	0.01	2.84	0.04	2.94	0.10	0.842	0.35
VSI (%)	5.44^a^	0.05	5.73^ab^	0.13	6.11^b^	0.09	6.07^b^	0.08	5.42^a^	0.02	0.000	16.93
HSI (%)	1.34^a^	0.01	1.34^a^	0.01	1.41^a^	0.02	1.56^b^	0.04	1.33^a^	0.06	0.002	9.10
IPF (%)	1.18	0.07	1.22	0.10	1.21	0.09	1.31	0.08	1.21	0.06	0.824	0.37

Data are reported as the mean and SEM (n = 3 for IBW, FBW, SGR, FE and Survival; n = 9 for CF, VSI, HSI and IPF). Values in the same line with different superscripts are significantly different (*P* < 0.05).

FBW, final body weight; FE, feed efficiency; IBG, initial body weight; SGR, specific growth rate.

CF, condition factor; HSI, hepatosomatic index; IPF, intraperitoneal fat ratio; VSI, viscerosomatic index.

### Biometric indices

The viscerosomatic (VSI) and hepatosomatic (HSI) indices were significantly higher in fish fed intermediate dietary DHA/EPA ratios (1.60 and 2.03), but fish fed the highest DHA/EPA ratio (2.67) showed no significant differences to fish fed the lowest DHA/EPA ratio (Table 4). In contrast, dietary DHA/EPA ratio had no effect on condition factor (CF) or intraperitoneal fat ratio (IPF) (*P* > 0.05).

### Whole body composition and tissue fatty acid compositions

Dry matter, crude protein and ash contents in the whole body did not show any statistical differences among the dietary treatments (Table 5). However, crude lipid level increased as the dietary DHA/EPA ratio increased from 0.65 to 1.16, and then decreased as the ratio increased further from 1.60 to 2.67.

**Table 5:** Whole body composition of the juvenile black seabream (*Acanthopagrus schlegelii*) (% wet weight) fed different dietary DHA/EPA ratios.

Parameter	Dietary DHA/EPA ratio											
	0.65		1.16		1.60		2.03		2.67		ANOVA *P*	F-value
	Mean	SEM	Mean	SEM	Mean	SEM	Mean	SEM	Mean	SEM		
Dry matter	30.14	0.18	30.54	0.10	30.13	0.21	29.99	0.37	30.37	0.68	0.84	0.35
Protein	18.51	0.07	18.47	0.13	18.32	0.28	18.27	0.12	18.19	0.46	0.89	0.27
Lipid	7.22^ab^	0.28	7.81^b^	0.05	7.73^b^	0.04	7.63^ab^	0.22	6.82^a^	0.26	0.03	4.27
Ash	4.89	0.04	4.66	0.04	4.68	0.07	4.70	0.09	4.95	0.13	0.08	2.87

Data are reported as means and SEM (n = 3). Values in the same line with different superscripts are significantly different (*P* < 0.05).

Liver and muscle fatty acid compositions reflected the dietary DHA/EPA ratios and thus as the dietary ratio increased, the DHA/EPA ratio in the tissues increased (Tables 6 and 7). The increasing dietary DHA/EPA ratio was achieved by a combination of increasing DHA and decreasing EPA (Table 2) and this was also reflected in the tissue compositions (Tables 6 and 7).

**Table 6:** Fatty acid compositions (% total fatty acids) of liver of juvenile black seabream (*Acanthopagrus schlegelii*) fed different dietary DHA/EPA ratios.

Name	Dietary DHA/EPA ratio											
	0.65		1.16		1.60		2.03		2.67		ANOVA *P*	F-value
	Mean	SEM	Mean	SEM	Mean	SEM	Mean	SEM	Mean	SEM		
14:0	2.69	0.11	2.62	0.10	2.81	0.06	2.82	0.11	2.76	0.01	0.52	0.87
16:0	15.56	0.36	15.06	0.23	15.40	0.39	15.65	0.34	15.10	0.20	0.60	0.72
18:0	14.32	0.54	13.95	0.37	14.12	0.08	14.35	0.10	13.34	0.56	0.41	1.10
∑SFA	32.57	0.97	31.64	0.63	32.33	0.36	32.81	0.17	31.20	0.41	0.32	1.36
16:1n-7	5.30	0.19	4.79	0.21	5.00	0.21	4.98	0.03	4.76	0.12	0.23	1.68
18:1n-9	25.89	1.32	25.56	0.50	24.65	0.66	24.65	0.31	23.83	0.98	0.47	0.96
∑MUFA	31.19	1.51	30.35	0.48	29.65	0.81	29.63	0.29	28.59	1.10	0.43	1.04
18:2n-6	6.95	0.10	6.91	0.27	6.49	0.01	6.20	0.32	6.62	0.19	0.15	2.18
20:2n-6	4.12	0.12	4.16	0.13	4.03	0.18	3.90	0.28	4.05	0.24	0.90	0.25
20:3n-6	1.48^a^	0.08	1.60^ab^	0.06	1.68^ab^	0.01	1.62^ab^	0.09	1.77^b^	0.03	0.07	3.13
20:4n-6	3.83	0.22	4.10	0.16	4.23	0.18	4.17	0.25	4.49	0.25	0.36	1.22
22:5n-6	0.27^a^	0.11	1.13^b^	0.11	1.60^c^	0.05	1.87^c^	0.07	2.33^d^	0.11	0.00	71.05
∑n-6PUFA	16.65^a^	0.34	17.90^ab^	0.51	18.04^ab^	0.21	17.76^ab^	0.43	19.27^b^	0.31	0.01	6.24
18:3n-3	0.29	0.03	0.43	0.06	0.31	0.03	0.29	0.02	0.41	0.04	0.06	3.19
20:5n-3(EPA)	2.48^b^	0.18	2.22^b^	0.10	1.79^a^	0.06	1.47^a^	0.04	1.43^a^	0.10	0.00	17.83
22:5n-3	2.42	0.68	1.96	0.52	1.88	0.46	1.77	0.26	1.64	0.23	0.80	0.41
22:6n-3(DHA)	4.81^a^	0.46	5.81^ab^	0.45	6.19^ab^	0.05	6.75^b^	0.49	7.23^b^	0.46	0.02	4.97
∑n-3PUFA	9.99	1.31	10.42	0.62	10.17	0.49	10.29	0.63	10.71	0.83	0.98	0.11
n-3/n-6 PUFA	0.60	0.07	0.58	0.05	0.56	0.04	0.58	0.05	0.55	0.03	0.97	0.13
n-3 LC-PUFA	9.70	1.29	9.99	0.68	9.87	0.47	9.87	0.47	10.30	0.79	0.99	0.07
DHA/EPA	1.94^a^	0.07	2.61^b^	0.15	3.46^c^	0.10	4.57^d^	0.23	5.05^d^	0.09	0.00	86.28

Data are reported as means and SEM (n = 3). Values in the same line with different superscripts are significantly different (*P* < 0.05). Some fatty acids in trace amount such as 12:0, 20:0, 22:0, 24:0, 20:1n−9, 22:1n−11, 16:2n−6, 18:3n−6, 18:4n−3, 20:3n−3 and 20:4n−3 were not listed. DHA/EPA, 22:6n−3/20:5n−3; long-chain PUFA (C_20-24_); MUFA, monounsaturated fatty acids; PUFA, polyunsaturated fatty acids; SFA, saturated fatty acids.

**Table 7:** Fatty acid compositions (% total fatty acids) of muscle of juvenile black seabream (*Acanthopagrus schlegelii*) fed different dietary ratios of DHA/EPA.

Name	Dietary DHA/EPA ratio											
	0.65		1.16		1.60		2.03		2.67		ANOVA *P*	F-value
	Mean	SEM	Mean	SEM	Mean	SEM	Mean	SEM	Mean	SEM		
14:0	1.83^a^	0.19	2.16a^b^	0.08	2.31^b^	0.09	2.33^b^	0.08	2.31^b^	0.03	0.04	3.97
16:0	18.52	0.13	19.15	0.91	19.44	0.69	19.04	0.30	19.30	0.26	0.79	0.43
18:0	9.17	0.04	8.95	0.06	8.95	0.25	8.90	0.07	8.83	0.19	0.57	0.77
∑SFA	29.52	0.26	30.26	0.80	30.71	0.53	30.27	0.30	30.44	0.24	0.52	0.87
16:1n-7	3.50	0.13	3.57	0.16	3.55	0.10	3.72	0.11	3.30	0.06	0.22	1.73
18:1n-9	19.51	0.35	20.12	0.39	20.05	0.45	19.54	0.51	18.49	0.31	0.11	2.53
∑MUFA	23.00	0.43	23.69	0.23	23.61	0.56	23.26	0.41	21.79	0.26	0.04	3.74
18:2n-6	9.38	0.24	9.63	0.40	9.67	0.14	9.58	0.14	9.51	0.04	0.91	0.24
20:2n-6	1.89	0.13	2.01	0.07	1.84	0.05	1.94	0.08	1.76	0.08	0.35	1.25
20:3n-6	1.45	0.10	1.38	0.01	1.39	0.07	1.52	0.02	1.34	0.08	0.40	1.11
20:4n-6	5.83	0.28	5.43	0.20	5.38	0.33	5.50	0.23	5.86	0.14	0.52	0.87
22:5n-6	0.95^a^	0.05	2.26^b^	0.13	2.89^c^	0.09	3.52d	0.16	4.09^e^	0.05	0.00	128.42
∑n-6PUFA	19.50^a^	0.24	20.70^ab^	0.48	21.17^ab^	0.57	22.05^b^	0.51	22.56^b^	0.25	0.00	7.68
18:3n-3	0.78	0.06	0.91	0.12	0.82	0.03	0.81	0.02	0.86	0.01	0.62	0.69
20:5n-3 (EPA)	5.21^c^	0.13	3.70^b^	0.34	3.27^b^	0.17	3.52^b^	0.16	2.50^a^	0.06	0.00	25.73
22:5n-3	2.56^b^	0.13	1.87^a^	0.03	1.73^a^	0.17	1.81^a^	0.16	1.39^a^	0.09	0.00	11.42
22:6n-3 (DHA)	9.76^a^	0.21	9.46^a^	0.38	10.46^a^	0.41	10.81^a^	0.37	12.46^b^	0.19	0.00	13.22
∑n-3PUFA	18.31^b^	0.24	15.94^a^	0.35	16.28^a^	0.72	16.95^ab^	0.39	17.21^ab^	0.26	0.02	4.56
n-3/n-6 PUFA	0.94^b^	0.02	0.77^a^	0.02	0.77^a^	0.02	0.77^a^	0.01	0.76^a^	0.01	0.00	22.39
n-3 LC-PUFA	17.52^b^	0.18	15.03^a^	0.26	15.47^a^	0.70	16.14^ab^	0.37	16.35^ab^	0.26	0.01	5.75
DHA/EPA	1.88^a^	0.09	2.62^b^	0.36	3.21^b^	0.12	3.07^b^	0.04	4.99^c^	0.17	0.00	36.65

Data are reported as means and SEM (n = 3). Values in the same line with different superscripts are significantly different (*P* < 0.05). Some fatty acids in trace amount such as12:0, 20:0, 22:0, 24:0, 20:1n−9, 22:1n−11, 16:2n−6, 18:3n−6, 18:4n−3, 20:3n−3 and 20:4n−3 were not listed. DHA/EPA, 22:6n−3/20:5n−3; long-chain PUFA (C_20-24_); MUFA, mono-unsaturated fatty acids; PUFA, polyunsaturated fatty acids; SFA, saturated fatty acids.

### Oxidation and antioxidant parameters

The activities of the antioxidant enzymes SOD (units/ml and units/mg protein) and GSH-PX (units/ml and units/mg protein) as well as the levels of GSH (mg GSH/L and mg GSH/g protein) in both serum and liver were not impacted by dietary DHA/EPA ratio (Table 8). In contrast, the level of MDA (nmol/ml and nmol/mg protein) in serum increased as dietary DHA/EPA increased from 0.65 up to 2.03, with no further increase observed beyond that point (Table 8). The level of MDA in liver showed a similar trend although this was not statistically significant (Table 9).

**Table 8:** Serum and liver oxidation and antioxidant parameters of juvenile black seabream (*Acanthopagrus schlegelii*) fed different dietary DHA/EPA ratios.

Parameter	Dietary DHA/EPA ratio											
	0.65		1.16		1.60		2.03		2.67		ANOVA *P*	F-value
	Mean	SEM	Mean	SEM	Mean	SEM	Mean	SEM	Mean	SEM		
Serum												
GSH-PX (units/ml)	23.61	1.21	25.91	0.80	23.75	0.20	23.31	1.69	22.93	1.27	0.63	0.68
SOD (units/ml)	77.48	4.14	81.51	4.15	91.96	8.00	85.37	6.48	83.69	3.89	0.49	0.92
GSH (mg GSH/L)	39.57	2.89	38.57	1.62	41.90	1.59	36.33	4.47	37.65	6.01	0.86	0.32
MDA (nmol/ml)	10.38^a^	1.36	10.38^a^	0.89	10.69^a^	0.98	15.50^b^	0.49	15.07^b^	0.28	0.00	8.90
Liver												
GSH-PX (units/mg protein)	181.43	13.81	203.68	16.25	232.30	12.94	218.65	17.09	207.02	7.96	0.20	1.83
SOD (units/mg protein)	77.92	3.84	70.37	4.55	82.25	7.57	76.41	4.42	75.38	3.53	0.59	0.74
GSH (mg GSH/g protein)	9.54	0.71	9.37	0.67	9.02	0.31	9.65	0.66	10.03	0.09	0.76	0.46
MDA (nmol/mg protein)	1.85	0.16	1.95	0.11	1.98	0.10	1.98	0.07	2.51	0.25	0.08	2.88

Data are reported as means and SEM (n = 3). Values in the same line with different superscripts are significantly different (*P* < 0.05).

GSH, glutathione; GSH-PX, glutathione peroxidase; MDA, malondialdehyde; SOD, superoxide dismutase.

**Table 9:** Hematological parameters (include the serum and the whole blood) of juvenile black seabream (*Acanthopagrus schlegelii*) fed different dietary DHA/EPA ratios.

Parameter	Dietary DHA/EPA ratio											
	0.65		1.16		1.60		2.03		2.67		ANOVA *P*	F-value
	Mean	SEM	Mean	SEM	Mean	SEM	Mean	SEM	Mean	SEM		
Serum												
Total protein (g/L)	39.43	0.85	38.53	2.13	37.03	1.71	37.80	2.00	36.33	1.43	0.72	0.52
Cholesterol (mmol/L)	5.26	0.22	4.80	0.18	5.21	0.08	5.18	0.03	5.25	0.11	0.21	1.80
TAG (mmol/L)	3.67^b^	0.13	3.09^a^	0.01	2.78^a^	0.18	2.90^a^	0.05	2.91^a^	0.11	0.00	10.14
Glucose (mmol/L)	7.00	0.48	6.82	0.18	6.57	1.01	7.59	0.55	6.50	0.30	0.69	0.57
Whole blood												
WBC (*10^9^/L)	105.77	4.76	107.33	6.93	105.57	3.48	104.75	1.41	104.73	6.38	1.00	0.05
RBC (*10^12^/L)	3.67	0.16	3.88	0.26	3.72	0.21	3.67	0.03	3.46	0.11	0.56	0.78
HGB (g/L)	103.67	4.48	101.50	1.44	109.50	4.63	103.67	3.84	102.50	2.02	0.94	0.19
HCT	0.47	0.03	0.46	0.02	0.53	0.03	0.50	0.03	0.56	0.03	0.09	2.73

Data are reported as means and SEM (n = 3). Values in the same line with different superscripts are significantly different (*P* <0.05).

HCT, hematocrit; HGB, hemoglobin; RBC, red blood cell; TAG, triacylglycerol; WBC, white blood cell.

### Hematological parameters

Serum TAG (mmol/L) level was reduced by higher dietary DHA/EPA ratios with the level in fish fed the low ratio (0.65) having significantly higher serum TAG than fish fed the higher ratios (Table 9). In contrast, dietary DHA/EPA ratio had no effect on serum protein, cholesterol or glucose levels. Furthermore, dietary DHA/EPA ratio did not influence WBC (*10^9^/L) or RBC (*10^12^/L) counts, or HGB (g/L) level and, although there was a trend for increasing HCT, this was not significant.

### Expression of lipid metabolism genes

**Fig 1:**
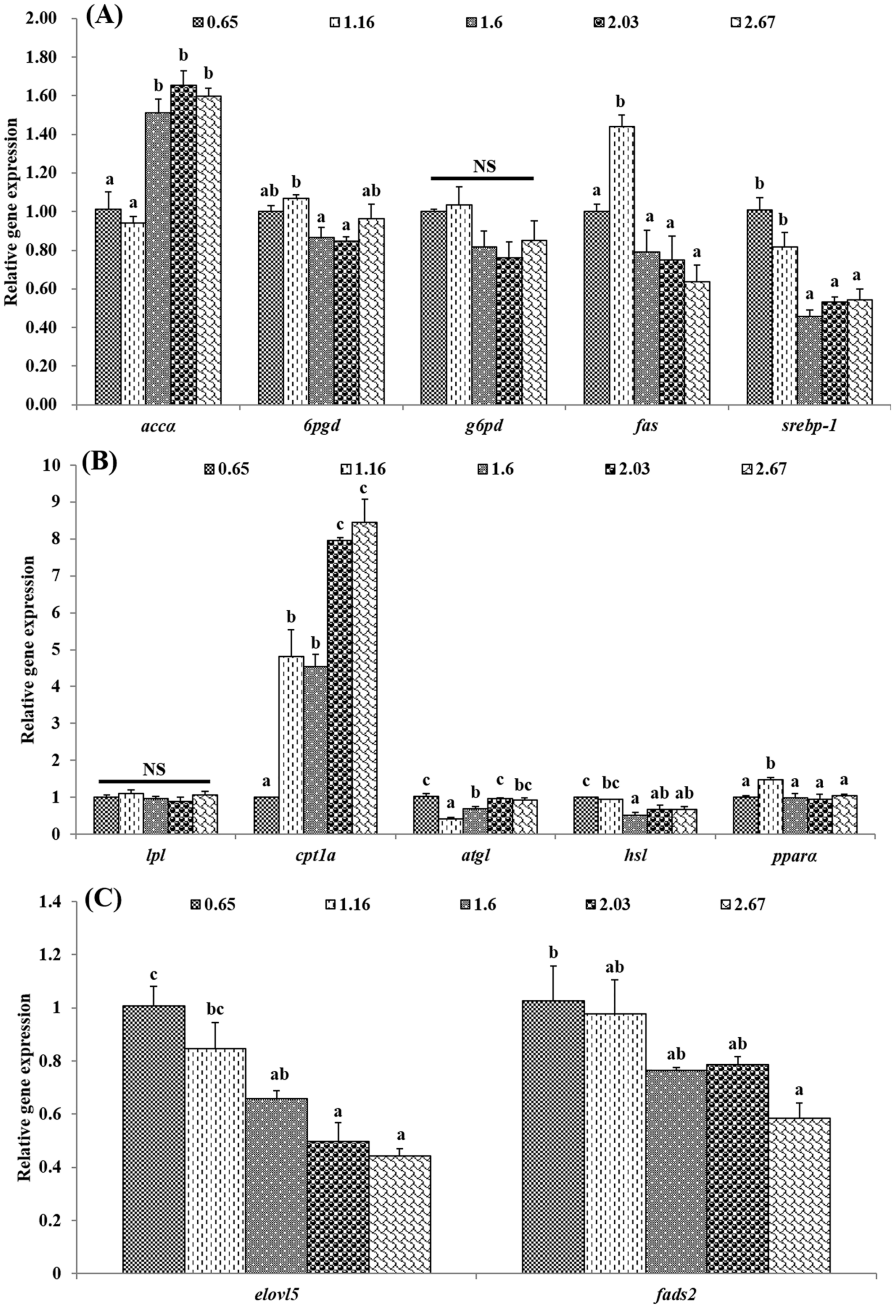
Effects of dietary DHA/EPA ratio on relative mRNA expression of genes involved in lipid metabolism pathways including anabolism (A), catabolism (B) and LC-PUFA biosynthesis (C) in the liver of juvenile black seabream (*Acanthopagrus schlegelii*). The control group (0.65 DHA/EPA) was used as the reference group, and the mRNA expression levels of target genes were normalized relative to the expression of *β-actin*. Values are means (n = 3), with standard errors represented by vertical bars. Mean values for the same gene with different letters were significantly different (*P* < 0.05). *accα*, acetyl-CoA carboxylase alfa; *6pgd*, 6-phosphogluconate dehydrogenase; *g6pd*, glucose 6-phosphate dehydrogenase; *fas*, fatty acid synthase, *srebp-1*, sterol regulatory element-binding protein-1; *lpl*, lipoprotein lipase; *cpt1a*, carnitine palmitoyltransferase 1A; *atgl*, adipose triglyceride lipase; *hsl*, hormone-sensitive lipase; *pparα*, peroxisome proliferator-activated receptor alpha; *fads2*, fatty acyl desaturase 2 and *elovl5*, elongase of very long-chain fatty acids 5. NS, not significant.

The expression of genes related to several lipid metabolism pathways including anabolism (panel A), catabolism (panel B) and LC-PUFA biosynthesis (panel C) in the liver of black seabream juveniles is shown in Fig 1. Among genes related to lipid biosynthesis pathways, the dietary DHA/EPA ratio significantly affected the expression levels of *accα*, *6pgd*, *fas* and *srebp-1* (Fig 1A). The liver expression level of *accα* increased significantly with increase dietary DHA/EPA ratios of 1.60 or beyond, with an opposite trend, i.e. down-regulation with increasing DHA/EPA ratios, observed for *srebp-1*. Transcript levels of *6gpd* in the liver of juvenile black seabream fed diets with intermediate DHA/EPA ratios (1.16, 1.60 and 2.03) were significantly higher than those of fish fed diets with lower (0.65) and higher (2.67) DHA/EPA ratios. The highest expression level of *fas* was observed in fish fed the diet with a dietary DHA/EPA ratio of 1.16.

With regards to genes related to lipid catabolism, the expression levels of *cpt1a*, *atgl*, *hsl* and *pparα* were all significantly affected by dietary DHA/EPA ratio (Fig 1B). The expression of *cpt1a* significantly increased with increased dietary DHA/EPA ratios of 1.16 and over. The lowest expression levels of *atgl* and *hsl* were found in fish fed the diets containing DHA/EPA ratios of 1.16 and 1.60, respectively, with high expression levels obtained in fish fed the lowest dietary DHA/EPA ratio (0.65). In contrast, *pparα* expression increased as dietary DHA/EPA ratio increased from 0.65 to 1.16 and then decreased with further increases in the ratio.

Dietary DHA/EPA ratio had a clear impact on the genes encoding the enzymes of LC-PUFA biosynthesis, with both *elovl5* and *fads2* showing graded decreased expression with increasing dietary DHA/EPA ratio (Fig 1C).

## Discussion

Several studies have demonstrated a higher biological value (e.g. enhancing growth performance and immunity) for DHA than for EPA during first feeding of marine fish species such as red seabream (*Pagrus major*), gilthead seabream (*Sparus aurata* L.) and turbot (*Scophthalmus maximus*) [51–55]. These results suggested that n-3 LC-PUFA requirements might not only be a function of the total amount of these fatty acids in the diet, but also of the relative proportions of essential LC-PUFA like DHA and EPA [56]. However, the results of the present study indicated that dietary DHA/EPA ratios within the tested dietary range (0.65–2.67) had no significant impact on growth performance or feed utilization of juvenile black seabream. These results are somewhat contradictory with those of a recent study on juveniles of large yellow croaker (*Larmichthys crocea*), where it was reported that high dietary ratios of DHA/EPA (2.17–3.04) significantly improved growth performance [45]. While the reasons for such an apparent discrepancy cannot be established, it cannot be ruled out that dietary DHA/EPA ratio above 2.6 could have also resulted in growth enhancing effect in black seabream juveniles. More clearly though, the present study revealed that dietary DHA/EPA ratio has impacts on body composition (also including fatty acid composition), oxidation and antioxidant parameters, hematological characteristics and lipid metabolism gene expression of genes involved in lipid and fatty acid metabolism was also studied.

Significant differences were found in VSI and HSI, with highest values in fish fed intermediate DHA/EPA ratios (1.60–2.03) and lower values found in fish fed both the lowest and highest DHA/EPA ratios. Since high HSI is associated with high energy reserves and metabolic activity [57], these results indicated that fish fed diet with intermediate DHA/EPA ratios were in a good nutritional status. A similar trend was found in the lipid content of whole body with fish fed intermediate DHA/EPA ratios having the highest levels of lipid. Body fat deposition may be influenced by the levels of endogenous lipid synthesis or catabolism or a combination of both mechanisms. The *atgl*, *hsl*, *fas* and *6pgd* genes encode important enzymes involved in mechanisms of lypolysis and lipogenesis [29]. The gene expression or activity of lipogenic enzymes, including Fas (*fas*), Accα (*accα*) and 6pgd (*6pgd*) have been described previously [58–62] and, likewise, studies have indicated that gene expression of lipolytic enzymes such as Atgl (*atgl*) and Hsl (*hsl*) were regulated by dietary modifications [29,62–67]. In the present study, the relative expression of *atgl* and *hsl* showed similar trends, with higher expression levels in fish fed the lowest DHA/EPA ratio (0.65) and lower expression in fish fed intermediate ratio (1.16). In contrast, *6pgd* showed the opposite pattern. The *accα* and *fas* genes play important roles in fatty acid biosynthesis [21] and, in the present study, the expression of *accα* gene, which encodes the enzyme responsible for the production of malonyl-CoA, a key early step in the biosynthesis of fatty acids [19,20], increased with increased dietary DHA/EPA ratio. However, subsequent steps in fatty acid synthesis are catalyzed by the *fas* gene product [19,24] and, conversely, *fas* gene expression was reduced in fish fed the higher DHA/EPA ratios. Therefore, although there are conflicting data, the gene expression results generally confirmed the above results for HSI, VSI and whole body lipid content, and showed that lipogenesis and lipolysis might be directly related to the expression levels of *atgl*, *hsl*, *fas* and *6pgd* in black seabream.

The fatty acid compositions of liver and muscle of black seabream showed similar results, largely reflecting the fatty acid compositions of the diets. For example, the level of DHA and the DHA/EPA ratio were significantly increased with increased dietary DHA/EPA ratio in both liver and muscle. On the contrary, EPA levels of liver and muscle decreased significantly with increased dietary DHA/EPA ratio, in agreement with a previous study in large yellow croaker using similar dietary formulations [57]. Many studies have reported that Fads2 and Elovl5 are two key enzymes in the LC-PUFA biosynthesis pathways [1,68–69]. In the present study, fish fed the lowest dietary DHA/EPA ratio (0.65) showed significantly higher expression of both *fads2* and *elovl5*. Consistently, the *fads2* expression of large yellow croaker also increased with decreasing dietary DHA/EPA [69] and moreover, these results are in agreement with the effect that DHA had on down-regulating *fads2* and *elovl5* in rainbow trout (*Oncorhynchus mykiss*) [70]. However, the increased expression of *fads2* and *elovl5* observed in the present study for low DHA/EPA dietary treatments did not resulted in increased enzymatic activity to compensate for lower dietary input. This is in agreement with a study on gilthead seabream in which tissue fatty acid profiles did not reflect up-regulation of *fads2* in fish fed vegetable oil [71]. Overall, this clearly indicates that diet is a major factor determining tissue fatty acid profiles in comparison to LC-PUFA endogenous production (biosynthesis).

Lipid peroxidation is caused by free radicals leading to oxidative destruction of PUFA constitutive of cellular membranes [72]. Evidence of lipid peroxidation in the form of increased MDA production, a surrogate marker of oxidative stress, has been noted [73]. In the present study, the serum MDA level was increased with increased dietary DHA/EPA ratio, and liver MDA level showed a similar trend. These results indicated that high dietary DHA/EPA ratio might induce higher oxidative stress in juvenile black seabream. Previous studies demonstrated that excess PUFA in liver may lead to increased lipid peroxidation [22,74–75]. The defenses against free radical-mediated injury include enzymatic deactivation and direct reaction with free radicals, such as SOD and GPX [73,76]. SOD is the first line of defense against oxygen derived free radicals and GPX catalyzes reductive destruction of hydrogen and lipid hydroperoxides, using GSH as an electron donor [36–39]. Furthermore, GSH is the most abundant non-enzymatic antioxidant present in the cell, plays an important role in the defense against oxidative-stress-induced cell injury [72]. However, in our study, the activities of SOD and GPX, as well as the content of GSH both in the liver and serum, showed no significantly different among groups. In this study, we demonstrated that higher DHA/EPA ratios could cause higher oxidative stress level, but could not enhance the antioxidant defense capability, at least through the activation of SOD and GSH-PX measured in this study.

β-oxidation is regulated mainly by the transcription factor *ppara*, and it regulates the expression of genes that participate in fatty acid oxidation, for instance, the *cpt1a* [77–78]. Cpt1a is regarded as the main regulatory enzyme in fatty acid β-oxidation [19,27]. Previous studies demonstrated that mitochondrial β-oxidation can be inhibited by reducing the activity of CptI [75, 79–80], and lipid accumulation mainly occurred because the excess lipids that were consumed could not be oxidized [75]. In this study, although, the expression level of *pparα* increased significantly to the maximum levels with dietary ratio of DHA/EPA increased from 0.65 to 1.16 and then decreased significantly with further increased of dietary DHA/EPA ratio. However, the highest expression level of *cpt1a* and the lowest lipid content were all recorded in fish fed the highest DHA/EPA ratio. Therefore, our study indicated that fatty acid oxidation is increased by up-regulating *cpt1a*, therefore resulting in reduced lipid content as previously reported elsewhere [75,79–80]. We speculate that dietary DHA/EPA ratio could affect the gene relative expression of *cpt1a*, and then influenced the fatty acid oxidation in black seabream.

Although there are relatively few data on the effects of dietary DHA/EPA ratio on hematological characteristics of marine fish, it has been demonstrated that they can be affected by diet and thus be good indicators of nutrition, stress, and the overall health of fish [81–82]. Zhou *et al*. [10] stated that RBC are both mechanical and biochemical barriers against infections, bacteria, and blood parasites and immune reactions are regulated to ensure harmony between the RBC and WBC populations. In addition, HGB in aquatic animals operates over wide and independent variations in oxygen at the sites of loading and unloading and shows adaptations both to environmental conditions and metabolic requirements, which govern oxygen availability and transport to tissues [10,83]. In the present study, however, the whole blood indices (WBC, RBC, HGB and HCT) were not affected by dietary DHA/EPA ratio suggesting that this dietary parameter did not affect fish health condition.

We herein observed that *srebp-1* expression decreased with increased dietary DHA/EPA ratio, whereas expression of *lpl* was not affected by the ratio. *Srebp-1* is a transcription factor regulating fatty acid and lipid biosynthesis pathways [19,84] and *lpl* hydrolyzes TAG in plasma lipoproteins providing fatty acids for storage in adipose tissue [19,26]. Serum TAG was highest in fish fed the diet with the lowest DHA/EPA ratio (0.65) whereas serum cholesterol was unaffected by the ratio. Yan et al [79] suggested that higher gene expression of *cpt1* might reduce the content of TAG, similar result was obtained in this current study. Few data are available on the effects of dietary DHA/EPA ratio on serum indices and, therefore, further studies are required to better understand the regulation of indices related to lipid metabolism in black seabream. There is evidence in both fish and humans that serum cholesterol might be influenced by dietary n3-LC-PUFA level [74,85–90], although, in the present study, the overall dietary n-3-LC-PUFA level was essentially the same in all diets. From the above, we suggest that increased dietary DHA/EPA ratio (with constant dietary n-3 LC-PUFA) may reduce serum TAG, but with no impact on serum cholesterol and further investigations are required.

In conclusion, the present study showed that although the dietary ratio of DHA/EPA did not affect growth performance or feed utilization, it did impact tissue fatty acid profiles, antioxidant capacity, hematological characteristics and expression of lipid related genes in juvenile black seabream. The study is the first to measure lipid anabolism and catabolism genes to explore mechanisms related to the physiological effects of dietary DHA/EPA ratio in black seabream.
